# Synergistic Potential of Plant Alkaloids and Intragenic Antimicrobial Peptides in Treating Multidrug-Resistant Infectious Diseases

**DOI:** 10.3390/antibiotics15060561

**Published:** 2026-05-31

**Authors:** Athamy Sarah de Paula Cruz, Thaís Campos de Sousa, Natália Elisabeth Kruklis, Nilton Araripe dos Santos Neto, Bianca Oliveira do Vale Lira, Gabriel Rocha de Andrade, Octávio Luiz Franco, Guilherme Dotto Brand, Marcelo Henrique Soller Ramada

**Affiliations:** 1 Programa de Pós-Graduação em Ciências Genômicas e Biotecnologia, Universidade Católica de Brasília, Brasilia 71966-700, DF, Brazil; athamysarah@gmail.com (A.S.d.P.C.); s.thaiscampos@gmail.com (T.C.d.S.); kruklisnatalia.24@gmail.com (N.E.K.); biancavalelira@gmail.com (B.O.d.V.L.); andraderochagabriel@gmail.com (G.R.d.A.); ocfranco@gmail.com (O.L.F.); 2 Programa de Pós-Graduação em Patologia Molecular, Universidade de Brasília, Brasilia 70910-000, DF, Brazil; niltonararipeneto@gmail.com; 3 S-Inova Biotech, Programa de Pós-Graduação, Universidade Católica Dom Bosco, Campo Grande 79117-900, MS, Brazil; 4 Laboratório de Síntese e Análise de Biomoléculas, Instituto de Química, Universidade de Brasília, Brasilia 70910-000, DF, Brazil; gdbrand@unb.br; 5 Programa de Pós-Graduação em Gerontologia, Universidade Católica de Brasília, Brasilia 71966-700, DF, Brazil

**Keywords:** antimicrobial resistance, intragenic antimicrobial peptides, alkaloids, synergistic combinations, biofilm inhibition

## Abstract

Background: Nosocomial infections caused by multidrug-resistant microorganisms are a significant public health concern. Antimicrobial resistance (AMR) is closely linked to the excessive and indiscriminate use of antibiotics, which creates selective pressure and promotes the emergence of resistant pathogens. Objectives: This study evaluates the synergistic potential of intragenic antimicrobial peptides (IAPs) combined with plant alkaloids against susceptible and multidrug-resistant human pathogenic bacteria, assessing antimicrobial activity, biofilm inhibition, and hemocompatibility. Methods: The tested molecules included berberine, tomatidine, sinomenine, and the IAPs Hs02 and Gr01. Minimum inhibitory concentration (MIC) and minimum microbicidal concentration (MMC) assays were performed against both ATCC (*E. coli* ATCC 25922 and *S. aureus* ATCC 25923) and clinical strains (*E. coli* KPC+ HRAN 1812446 and *S. aureus* MDR LACEN 3730529). Synergistic interactions were evaluated by checkerboard assay, followed by biofilm inhibition and hemolysis assays using human red blood cells. Results: Berberine exhibited a MIC of 1024 µM when tested individually, while tomatidine and sinomenine showed no significant activity. As expected, the IAPs showed strong antimicrobial properties at 8 µM (Hs02) and 4 µM (Gr01). When tested in synergy, alkaloids and IAPs reduced the MIC by up to 128-fold. The combination of IAPs and alkaloids reduced the biofilm biomass of *S. aureus* and *E. coli* by 50%, by the crystal violet assay (*p* < 0.05). Notably, sinomenine had not previously been reported to have antimicrobial activity. Conclusions: These results highlight the importance of further exploring combinations of natural and synthetic bioactive molecules as promising antimicrobial candidates. This approach may help to extend the useful life of conventional antibiotics. However, further studies are needed to assess safety, cytotoxicity, genotoxicity, inflammation, and in vivo effects.

## 1. Introduction

The rapid escalation of antimicrobial resistance (AMR) poses a critical threat to global public health, as highlighted by the World Health Organization (WHO), which classifies MDR ESKAPE pathogens as a priority for developing new therapeutic strategies [1,2,3]. The conventional antibiotic pipeline is insufficient to keep pace with evolving resistance mechanisms in this scenario. Consequently, there is an urgent need for alternative approaches, such as using natural alkaloids and intragenic antimicrobial peptides (IAPs), which have multi-target action and the potential to restore the efficacy of existing drugs [4,5].

One of the most promising alternative strategies involves antimicrobial peptides (AMPs). These molecules have been widely investigated as potential substitutes for conventional antibiotics, as they are naturally produced by virtually all living organisms as part of the innate immune system. Building on the extensive characterization of peptide sequences encoded by genes from diverse biological sources reported in the literature, these molecules can be rationally modified to enhance their biological activity or to identify novel AMP candidates [6,7,8]. The physicochemical properties of AMPs can be used to identify similar peptide sequences within complex protein sequences. Once located and synthesized, these short sequences derived from the parent protein are known as intragenic antimicrobial peptides (IAPs) [9,10,11]. AMP-inspired IAPs are expected to have similar mechanisms of action to those of AMPs. Accordingly, they may exhibit broad-spectrum activity against Gram-positive and Gram-negative bacteria and yeasts [12,13]. Hs02, derived from the unconventional myosin 1h protein from *Homo sapiens* [11], and Gr01, derived from an uncharacterized protein LOC105765114 from *Gossypium raimondii* [10], are IAPs consisting of 16 amino acids (aa). Their sequences are KWAVRIIRKFIKGFIS and GFKLGRKLVKVFKWII, respectively. Both have broad-spectrum antimicrobial activity against Gram-positive and Gram-negative bacteria and fungi and showed better results than the reference antibiotics used as controls. Hs02 has previously been shown to have anti-inflammatory effects and to suppress TNF-alpha release by macrophages [11].

Alkaloids are a diverse class of natural metabolites found in many plant and animal species. They have notable antimicrobial, anti-inflammatory, and immunomodulatory properties. In addition to these effects, certain alkaloids have been reported to inhibit biofilm formation, modulate the expression of virulence factors, and interfere with resistance mechanisms, including bacterial efflux pumps [14,15]. Consequently, alkaloids emerge as a strategic class of compounds for overcoming antimicrobial resistance. Among the plant alkaloids, berberine, tomatidine, and sinomenine were selected for this study [16,17,18]. Berberine, isolated from the Berberidaceae family, is an isoquinoline alkaloid. It has been described as having antimicrobial, anti-inflammatory, and immunomodulatory activities (Th1, Th2, Th17, and Treg cells); antibiofilm activity against bacteria; inhibition of efflux pumps present in microbial membranes; and reported synergism activity with other molecules, such as antibiotics [14,19]. Some microorganisms have been reported to be susceptible to berberine, including *Enterococcus* sp., *Staphylococcus aureus*, *Acinetobacter baumannii*, *Pseudomonas aeruginosa*, *Klebsiella pneumoniae*, and *Mycobacterium avium* complex, both susceptible and resistant strains [20,21]. Combinations with berberine include antibiotics such as β-lactams, quinolones, aminoglycosides, tetracyclines, rifamycins, macrolides, lincosamides, and fusidic acid [21].

Tomatidine, a steroidal pseudo-alkaloid isolated from the Solanaceae family, has been reported to inhibit the replication of pathogenic bacteria, the growth of human-pathogenic microorganisms such as *S. aureus*, both susceptible and resistant strains, the hemolytic activity of certain strains, and the expression of virulence-associated genes. Tomatidine has also been described as an anti-inflammatory compound, with improvements in the inflammatory response associated with reduced serum levels of Th2-derived inflammatory cytokines [22,23,24]. Therefore, given the widely reported broad-spectrum and multi-target antimicrobial activities of berberine and tomatidine, as well as their ability to interfere with specific bacterial resistance mechanisms, such as efflux pumps, these compounds were strategically selected based on the literature.

Finally, sinomenine, isolated from the Menispermaceae family, is a morphinan alkaloid. Among the activities already described, it can reduce the expression of the cytokines IL-1α, IL-1β, IL-6, IL-10, IL-12, TNF-α, and the caspases 3 and 9, and is used to treat autoimmune diseases due to its anti-inflammatory and nociceptive actions [18,25,26,27]. In addition, sinomenine was chosen based on reported immunomodulatory properties, providing an added benefit in the treatment of complex infections.

Both alkaloids and peptides have considerable potential to exhibit synergistic activity with conventional antibiotics [28,29,30,31]. For example, Nisin is an AMP that has been shown to significantly reduce biofilm formation by *S. aureus* and *P. aeruginosa* [32], thereby increasing their susceptibility to conventional antibiotics. This effect is particularly significant in chronic infections, where biofilms act as physical and biochemical barriers to drug action. Furthermore, recent studies on the AMP Pt5-1c have demonstrated that combining it with antibiotics can reduce the minimum inhibitory concentration (MIC) by up to 16-fold, highlighting the potential of antimicrobial peptides, including IAPs, as therapeutic adjuvants [33].

These synergistic effects can improve activity against resistant organisms, prolong the useful life of antimicrobial formulations, and delay the need for new drug development. Consequently, such strategies reduce the required drug dosage during therapy, which can lower toxicity, shorten treatment duration, and, in turn, reduce the likelihood of further resistance development [34,35]. Thus, investigating these molecular mechanisms paves the way for more effective and sustainable therapies.

The synergism between alkaloids and antibiotics has also gained prominence. Previous investigation has demonstrated the efficacy of combining berberine with antibiotics against multidrug-resistant *Acinetobacter baumannii*, both potentiating antibiotic action and inhibiting efflux pumps [36].

Recent studies suggest that antimicrobial peptides (AMPs) and alkaloid-derived compounds may have complementary modes of action. From a theoretical perspective, these mechanistic differences provide a promising basis for synergistic therapies. Generally, AMPs are associated with membrane destabilization, pore formation, and increased cell permeability. In contrast, alkaloids are often associated with intracellular targets, including modulation of enzymatic activity, metabolic pathways, and DNA-related processes [37]. Although the cited study focuses on fungal infections, the underlying principles may also apply to bacteria, supporting the rationale for combining such compounds to target multiple cellular pathways and potentially enhance antimicrobial efficacy against resistant strains.

Therefore, IAPs and alkaloids are emerging as promising alternatives amid increasing antimicrobial resistance and the growing need for new therapeutic agents. The hypothesis is that combining IAPs and alkaloids is an innovative strategy to enhance antimicrobial activity against both ATCC strains and clinical isolates. These molecules, when used alone or in combination, have the potential to overcome the limitations of conventional antibiotics.

## 2. Results

Peptides, alkaloids, and antibiotics (controls) were individually tested to determine their MIC and MMC values towards *S. aureus* ATCC 25923 (*S. aureus* ATCC), *S. aureus* MDR LACEN 3730529 (*S. aureus* MDR), *E. coli* ATCC 25922 (*E. coli* ATCC), and *E. coli* KPC+ HRAN 1812446 (*E. coli* KPC+), as shown in Table 1. Ampicillin was only effective against *S. aureus* ATCC, while gentamicin was effective against all strains, except for *E. coli* KPC+, which was more susceptible to amikacin. Peptides Hs02 and Gr01 showed antimicrobial activity against all ATCC and clinical isolates, while Hs02 displayed a lower MIC and MMC against the *S. aureus* MDR when compared to the *S. aureus* ATCC.

DMSO assays showed an MIC at 8% (*v*/*v*) and an MMC between 16% and 32%. Consequently, all alkaloid assays were normalized so that the final DMSO concentration in each well did not exceed 2% (*v*/*v*), even at the highest tested alkaloid concentration. This ensured that the methodology remained safely below the solvent’s inhibitory and microbicidal thresholds. Among the tested alkaloids, only berberine demonstrated a MIC at the highest evaluated concentration (1024 µM for *S. aureus* ATCC and 512 µM for *S. aureus* MDR). Tomatidine and sinomenine showed no measurable MIC, and no MMC was observed for any alkaloid, even at the highest concentration tested.

The lack of a MIC for sinomenine was anticipated, as this alkaloid is primarily known for its immunomodulatory, rather than antimicrobial, activity. Since the MIC values for tomatidine and sinomenine were not reached within the tested concentration range, their actual MICs are presumed to be higher than 1024 µM. Therefore, to calculate the FICI for synergy assays, a theoretical MIC of 1024 µM was adopted for both tomatidine and sinomenine as a conservative estimate.

Synergy assays were then performed between the alkaloids and peptides. Table 2 summarizes the new MIC values for Hs02 and alkaloids in combination, along with the corresponding FICI values against ATCC and clinical isolates. Additional synergistic and additive interactions were also identified (Appendix A). Although peptide Gr01 exhibited MIC activity on its own, it showed limited synergistic or additive effects in combination assays (Appendix A).

It is noteworthy that even though the theoretical MIC of 1024 µM does not represent the real MIC for these alkaloids, the theoretical synergic FICI value (≤0.5) obtained in this scenario is higher when compared to the real FICI value, which would be even lower. Therefore, it is important to validate the MIC values for both alkaloids to obtain the corresponding FICI values and evaluate whether any additive or potentiating effects observed in Table 2 and Appendix A are synergistic.

**Figure 1:**
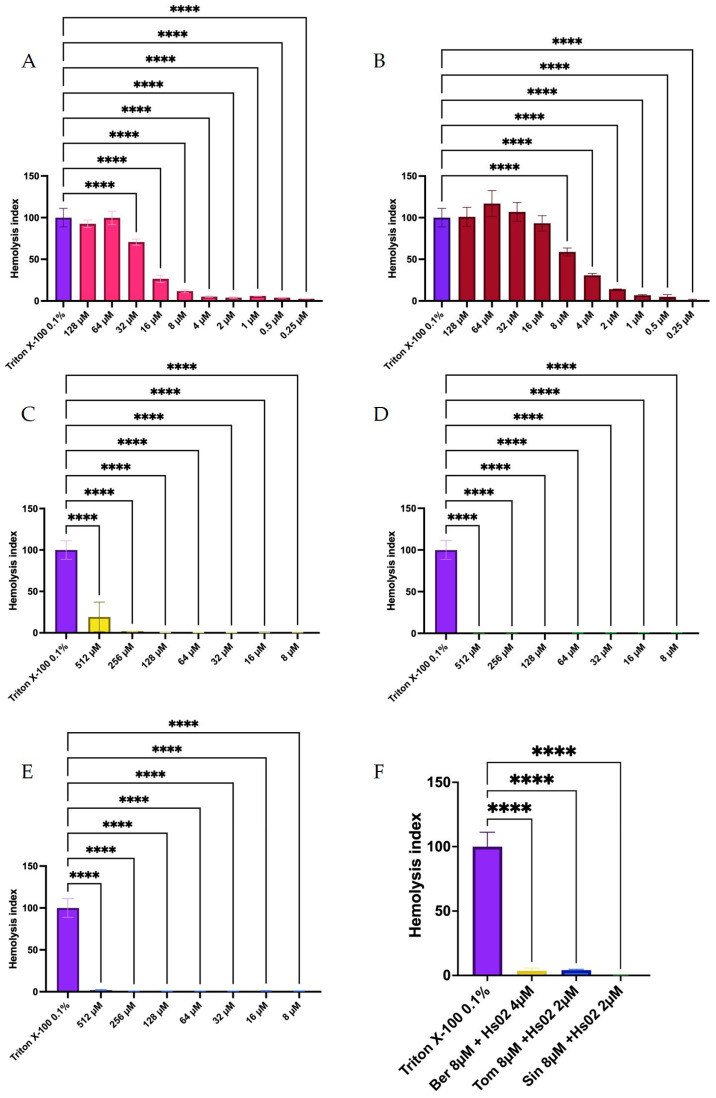
Hemolytic Activity of Peptides and Alkaloids Isolated and in Combination: (**A**) Hs02 Peptide, (**B**) Gr01 Peptide, (**C**) Berberine, (**D**) Sinomenine, (**E**) Tomatidine, and (**F**) Hs02 Combined with Alkaloids. The positive control (Triton X-100 0.1%) induced nearly 100% hemolysis, whereas the tested combinations (Ber 8 µM + Hs02 4 µM yellow, Tom 8 µM + Hs02 2 µM blue, and Sin 8 µM + Hs02 2 µM green) exhibited significantly lower hemolytic activity. Statistical analysis revealed a significant difference (****, *p* < 0.0001) between all tested combinations and the control. Error bars represent the standard deviation of the mean. The *p*-values are available in Appendix B.

Given their activities, peptides and alkaloids were tested for their interactions with human red blood cells. Hs02 and the alkaloids, when tested individually, exhibited low hemolytic activity (Figure 1), suggesting potential suitability for combination therapy. In contrast, Gr01 demonstrated higher hemolytic activity. Consequently, subsequent assays focused on Hs02, which combined greater synergistic potential with a lower hemolytic profile, both alone and in combination with the alkaloids (Figure 1 and Appendix B).

Regarding the minimum microbicidal activity of the Hs02-alkaloid combinations (Table 3), the bacteriostatic or bactericidal behavior differed according to the strain. Against *S. aureus* ATCC, the synergistic combinations of Ber + Hs02 and Sin + Hs02 exhibited bacteriostatic activity, whereas Tom + Hs02 was bactericidal. Against *E. coli* ATCC, Ber + Hs02 and Sin + Hs02 both classified as synergistic and were bactericidal, while Tom + Hs02 was synergistic but bacteriostatic.

Synergistic effects in the clinical isolates were observed for Sin + Hs02 against *S. aureus* MDR, and for both Tom + Hs02 and Sin + Hs02 against *E. coli* KPC+. Table 2 shows the lowest FICI values for the combinations. It is important to note that the lowest FICI combinations of sinomenine and Hs02 exhibited synergic effects and bactericidal activity against both strains, as well as bactericidal activity for the tomatidine and Hs02 combination against *E. coli* KPC+. Classified as synergic, the observed enhancement of alkaloid activity reached up to 128-fold, particularly in combinations with sinomenine (Table 3). Nevertheless, these findings should be interpreted with caution, and further investigation is required.

**Figure 2:**
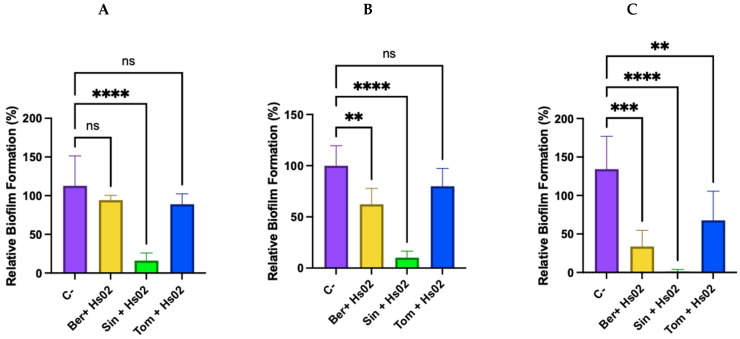
Bacterial biofilm formation inhibition. (**A**) *S. aureus* ATCC. (**B**) *S. aureus* MDR. (**C**) *E. coli* ATCC. Ber—berberine; Tom—tomatidine; Sin—sinomenine. The biofilm formations of *S. aureus* ATCC, *S. aureus* MDR, and *E. coli* ATCC were evaluated in the presence of different compound combinations (Ber 64 µM + Hs02 1 µM, yellow; Sin 8 µM + Hs02 2 µM, green, and Tom 8 µM + Hs02 1 µM, blue). The negative control (C−), purple, represents 100% biofilm formation. The tested combinations significantly reduced biofilm formation to varying degrees compared with the control. Statistical analysis revealed significant differences (*p* < 0.01, **; *p* < 0.001, ***; *p* < 0.0001, ****), while “ns” indicates non-significant differences. Error bars represent the standard deviation of the mean. The significance data, statistical difference index, and *p*-value are presented in Appendix C.

To further evaluate bacterial behavior in the presence of synergistic combinations with lower FICI values, a test was conducted to observe the prevention of bacterial biofilm formation. The results indicate that, for the *S. aureus* ATCC strain tested, only the sinomenine combination produced a statistically significant reduction in biofilm formation compared with the negative control (Figure 2 and Appendix C). However, in the analysis of multidrug-resistant *S. aureus*, a statistically significant difference was observed between the berberine–Hs02 and sinomenine–Hs02 combinations, with the latter showing a more pronounced effect. Across all tested strains, combinations with sinomenine consistently showed the greatest inhibition (*p* < 0.0001). Despite the observed reduction in biofilm biomass, these results are preliminary.

## 3. Discussion

The present study demonstrated that combinations of IAPs and plant-derived alkaloids significantly reduced the effective antimicrobial concentrations required against both ATCC and clinical isolates. Although tomatidine and sinomenine did not display antimicrobial activity when tested individually, their association with Hs02 led to marked reductions in the concentrations required to inhibit bacterial growth, including against resistant strains. Among the evaluated combinations, sinomenine-associated treatments showed particularly strong effects, suggesting that combining IAPs and alkaloids may be a promising strategy to enhance antimicrobial activity against clinically relevant pathogens.

Alkaloids are structurally diverse molecules derived from various plant metabolic pathways and are frequently associated with immunomodulatory, antimicrobial, and metabolic activities in humans and microorganisms [15]. The antimicrobial activity observed for berberine partially agrees with previous reports describing its effects against Gram-positive and Gram-negative bacteria, although the MIC values obtained in the present study were generally higher than those previously reported in the literature. Previous studies reported MIC values ranging from 148 to 496 µM for *S. aureus* [38] and values of 171.99 µM for *S. aureus* and 85.99 µM for *E. coli* [20], differing from the values observed in the present work, particularly for *E. coli*, in which no MIC was detected even at the highest concentration tested (1024 µM).

Similarly, tomatidine has previously been reported to inhibit *S. aureus* at low micromolar concentrations, including values around 3.85 µM [39], although other investigations also described MIC values above 308 µM for the same ATCC strains used in this study [40,41]. In contrast, the absence of detectable antimicrobial activity for sinomenine is consistent with the literature, which primarily characterizes this alkaloid as an immunomodulatory compound rather than a direct antimicrobial agent [42]. Together, these findings reinforce that the antimicrobial behavior of alkaloids is highly dependent on strain background, experimental setup, and physicochemical conditions.

The discrepancies observed between the MIC values obtained in this study and those reported in previous investigations may be partially explained by physicochemical interactions between the tested molecules and the cell culture-treated plates used during the assays. Unlike standard or low-binding microplates, these plates have modified surfaces that promote cellular adhesion and may adsorb peptides and alkaloids via electrostatic, hydrophobic, and van der Waals interactions. This effect is particularly relevant for cationic peptides, whose adsorption may become more pronounced at lower concentrations during serial dilution assays [43]. Consequently, the bioavailable concentration of the compounds in solution may have been reduced, as evidenced by the 8 µM MIC for Hs02 compared with the previously reported 1 µM [11], contributing to the higher MIC values observed. In addition, variations in MIC values may also reflect differences in bacterial phenotypes and resistance mechanisms among the strains evaluated in each study. Therefore, although the treated plates were selected to maintain consistency with future cell-based assays, this methodological aspect should be considered a limitation of the present study.

The clinical isolates also demonstrated distinct susceptibility profiles toward the evaluated molecules. Berberine exhibited a MIC of 512 µM against the multidrug-resistant *S. aureus* strain, which is partially consistent with previous reports involving resistant strains such as MRSA N315 [20]. Additional investigations evaluating berberine against resistant *S. aureus* strains reported MIC values ranging from 190.27 µM to 761.08 µM depending on the strain evaluated [44], reinforcing the importance of strain-specific responses in antimicrobial assays.

Regarding the antibiotic susceptibility results, the profiles observed for the reference strains *E. coli* ATCC 25922 and *S. aureus* ATCC 25923 (Table 1) were generally consistent with previously reported MIC ranges for gentamicin [45,46]. According to revised aminoglycoside breakpoints, Enterobacterales are considered resistant to gentamicin at MIC values ≥ 8 µg/mL and to amikacin at MIC values ≥ 16 µg/mL, whereas *Staphylococcus* spp. are considered resistant to gentamicin at MIC values ≥ 16 µg/mL [47,48]. The resistant strains exhibited the expected reduced susceptibility patterns, with MRSA showing high resistance to ampicillin, consistent with the characteristic resistance of methicillin-resistant *S. aureus* to β-lactam antibiotics [49,50]. In agreement with previous studies and established interpretive criteria, staphylococcal isolates with penicillin MIC values ≥ 0.25 µg/mL are considered resistant, a finding that also predicts resistance to aminopenicillins such as ampicillin [51,52,53]. Likewise, *E. coli* KPC+ displayed elevated MIC values, compatible with its multidrug-resistant phenotype associated with carbapenemase production [54,55]. The same trend was observed for amikacin, consistent with the literature [56] and the revised aminoglycoside breakpoints proposed by the CLSI.

Despite the limited antimicrobial activity observed for some alkaloids when tested individually, checkerboard assays showed reduced effective concentrations when combined with Hs02 (Table 2). Synergistic interactions were observed across several combinations, particularly against resistant strains, suggesting that these molecules may act cooperatively under the conditions tested. The particularly strong effect observed for the sinomenine-Hs02 combination against all strains is noteworthy because sinomenine had no previously established direct antimicrobial activity [16,42], making its behavior in combination with Hs02 biologically interesting and mechanistically informative.

Since experimentally determined MIC values were not reached for tomatidine and sinomenine within the evaluated concentration range, the FICI calculations involving these alkaloids were based on a theoretical MIC value of 1024 µM, corresponding to the highest concentration tested. Therefore, the resulting FICI values are conservative estimates and likely underestimate the true magnitude of synergistic interactions. Nevertheless, the marked reduction in effective concentrations observed during combination assays supports the biological relevance of these interactions.

Regarding antibiofilm activity, a prior study showed that only one of twelve *Clostridioides difficile* strains showed significant inhibition of biofilm formation upon treatment with berberine and sub-inhibitory vancomycin [57]. The present findings are partially consistent with this observation, though a direct comparison is constrained by differences in experimental context: vancomycin is a glycopeptide antibiotic structurally and mechanistically distinct from the IAP Hs02, and the bacterial species and strain backgrounds differ substantially. These factors likely contribute to the divergent outcomes and underscore the importance of strain- and molecule-specific evaluations in antibiofilm studies. In this study, biofilm inhibition by the berberine-Hs02 combination was not observed against *S. aureus* ATCC, but was statistically significant against the multidrug-resistant *S. aureus* strain (*p* = 0.0019) and *E. coli* ATCC (*p* = 0.0002). Despite its utility as a standard screening method, the crystal violet assay provides limited structural insight, and further characterization using imaging techniques such as confocal laser scanning microscopy (CLSM) or scanning electron microscopy (SEM) will be necessary to fully interpret the observed effects.

A previous report demonstrated that Tomatidine (144 µM) reduced biofilm formation, volume, and thickness in *L. monocytogenes*, likely by impairing cell–cell adhesion rather than directly inhibiting biofilm formation [58]. In contrast, no inhibitory effect was observed against *S. aureus* in the present study, despite both species being Gram-positive. On the other hand, biofilm formation by the Gram-negative *E. coli* ATCC was inhibited at 8 µM tomatidine and 1 µM Hs02, with a statistically significant difference compared to the control.

Sinomenine at a concentration of 9.1 μg/mL (27.63 µM) has been shown to reduce dispersion of *S. aureus* biofilms via cell–cell adhesion and disruption of mature biofilms, without inhibiting biofilm formation when used alone [59,60]. In the present study, sinomenine (8 µM) in combination with Hs02 (2 µM) appeared to inhibit the biofilm formation in all three tested strains, with statistically significant differences compared to the control (*p* < 0.0001). The shift from biofilm dispersion, observed at a higher concentration of sinomenine alone, to biofilm-formation prevention at a lower concentration in combination suggests that Hs02 might alter the conditions under which sinomenine exerts its activity. Considering that sinomenine is primarily characterized as an immunomodulatory compound rather than an antimicrobial agent [16], this antibiofilm effect is mechanistically intriguing and remains to be elucidated.

One plausible mechanistic hypothesis for the synergisms of Hs02 + alkaloids involves a two-step membrane-disruption model. Hs02 is known to act directly on bacterial membranes, inducing permeabilization and reducing membrane rigidity [11,61]. This initial membrane destabilization can lead to the disruption or penetration of these cellular structures. Berberine is a well-characterized DNA and RNA intercalator. Intercalation involves disrupting the molecular structure of nucleic acids by altering base stacking and interfering with bacterial protein synthesis [30]. Therefore, the ability of berberine to target genetic material, combined with the membrane-destabilizing effects promoted by Hs02, provides a plausible explanation for the synergistic interaction observed in the berberine–Hs02 checkerboard assays (FICI 0.18 and 0.5 for *S. aureus* ATCC and *E. coli* ATCC, respectively). This interpretation is consistent with the antibacterial performance of the combination, although the proposed mechanisms were not directly evaluated in the present study.

As for sinomenine, membrane destabilization induced by Hs02 may lower the energy barrier for passive diffusion of small amphiphilic molecules, thereby facilitating the intracellular accumulation of sinomenine at concentrations otherwise insufficient for bacteriostatic activity, leading to unprecedented synergism. Once inside the bacterial cell, sinomenine may interact with intracellular targets that remain to be identified. Unlike berberine, sinomenine lacks the planar polycyclic aromatic scaffold typical of DNA intercalators [30], and direct evidence of analogous DNA-targeting activity has not yet been reported. The precise intracellular mechanism underlying the observed synergism requires further investigation.

To support these hypotheses, an in silico analysis using SwissADME [62] was performed, revealing differences in lipophilicity among the evaluated alkaloids. Tomatidine exhibited high lipophilicity (consensus LogP = 4.60), whereas berberine (2.66) and sinomenine (1.99) exhibited moderate lipophilicity. All compounds presented low topological polar surface area (TPSA) values (<30 Å^2^), suggesting favorable membrane permeability. Notably, tomatidine exhibited a TPSA of 17.07 Å^2^, indicating its pronounced hydrophobicity. Although in silico predictions offer valuable insights into physicochemical properties, other in silico approaches, such as molecular dynamics simulations and experimental biophysical membrane interaction tests, are required to validate these parameters [63,64].

Intermediate lipophilicity is frequently linked with a more balanced solubility–permeability profile, which may impact antimicrobial efficacy. However, highly lipophilic compounds exhibit a greater affinity for lipid bilayers while showing reduced aqueous solubility [65]. This behavior was observed during the experimental phase of solution preparation, as berberine and tomatidine required DMSO to achieve adequate dissolution.

The hemolytic potential of the peptide Hs02, in combination with sinomenine, tomatidine, and berberine, was evaluated to assess its effects on erythrocyte membrane integrity. Conventional hemolysis assays generally employ erythrocyte concentrations ranging from 3% [66,67] to 4% [68,69]; however, this study deliberately utilized a 1% (*v*/*v*) suspension [70] to establish a more rigorous analytical condition. By reducing the cellular density, a significant increase in the molecule-to-cell ratio was achieved, thereby maximizing the probability of interaction between the tested compounds and the erythrocyte membranes. Consequently, this high-sensitivity approach guarantees that the observed lack of significant hemolysis is a robust indicator of the compounds’ hemocompatibility under acute exposure conditions.

Berberine exhibited mild cytotoxicity when tested alone at 512 µM (~30% cell death), likely due to its known narrow therapeutic window at higher concentrations [71,72]. In contrast, combinations of Hs02 with alkaloids showed no significant cytotoxicity and minimal hemolytic activity across the tested conditions. Although higher concentrations of berberine and IAPs individually induced greater hemolysis, their combination at relevant concentrations significantly reduced this effect, indicating low hemolytic activity under the tested conditions. Similar results were observed for Hs02 combined with sinomenine and tomatidine (Figure 2).

The low hemolytic activity exhibited by Hs02 at therapeutic concentrations suggests limited toxicity toward mammalian cells, with no significant damage to erythrocytes being observed [73]. This feature is especially important in the context of antimicrobial development, as many antimicrobial peptides are associated with substantial hemolysis and cytotoxicity. In the present study, the absence of significant hemolysis in Hs02 combinations, including those containing berberine, may be advantageous since lower effective peptide concentrations are required to achieve antimicrobial activity in synergistic formulations.

This low hemolytic profile is particularly relevant, given the potent antimicrobial effects previously reported for Hs02, including disruption of bacterial biofilms and reduced membrane rigidity in pathogens such as *P. aeruginosa* and *S. aureus* [61]. These activities are believed to result from direct interactions with bacterial membranes, driven by physicochemical properties that promote affinity for anionic membrane surfaces. Together, these findings highlight the potential of Hs02 as a promising candidate for antimicrobial therapies targeting resistant pathogens while maintaining limited toxicity toward mammalian cells.

Another study published peptide conjugates that target the synovium. These conjugates enhance the effect of sinomenine and minimize its systemic side effects [74]. As part of the characterization of these compounds, the hemolysis rate was evaluated to assess potential cytotoxic effects on red blood cells. The results showed that at therapeutic concentrations, sinomenine conjugates exhibited an insignificant hemolytic rate, suggesting a profile suitable for systemic administration. This finding reinforces the potential of sinomenine as an effective and safe therapeutic approach, provided it is formulated to optimize bioavailability and minimize interactions with cell membranes.

Similarly, the absence of significant hemolysis in Hs02-alkaloid combinations suggests that Hs02 can interact selectively with bacterial membranes at lower effective concentrations, preserving the integrity of human erythrocytes. Nevertheless, the increased cytotoxicity and hemolysis observed for IAPs and berberine at higher concentrations indicate a dose-dependent effect. Consistent with these observations, a previous study evaluating the safety profile of berberine demonstrated that although the compound can interact with cellular membranes, therapeutic doses did not induce significant hemolytic damage to red blood cells [75]. Together, these findings suggest that the hemolytic effects of these compounds can be minimized through controlled dosing and optimized combination strategies.

Taken together, these findings illustrate that peptide–alkaloid combinations can enhance antimicrobial efficacy against both reference and clinically relevant resistant strains, while maintaining a low hemolysis rate at the concentrations required for synergistic activity. The pronounced effect of the sinomenine–Hs02 combination is particularly noteworthy, given that sinomenine alone has no recognized direct antimicrobial activity, suggesting that this association may unveil a previously unexplored mode of action for an established immunomodulator. Although the present study did not directly address the underlying molecular events, the integrated evidence of synergistic FICI values, antibiofilm effects, low hemolytic activity, and physicochemical compatibility provides a coherent foundation for further mechanistic and translational investigations.

## 4. Material and Methods

### 4.1. Molecules and Microorganisms

Commercial antibiotics ampicillin, amikacin, and gentamicin were used; purified plant-derived alkaloids berberine, sinomenine, and tomatidine were purchased from Sigma-Aldrich (St. Louis, Mo, USA). The peptides Hs02 and Gr01 were synthesized by solid-phase synthesis using the F-moc strategy [76] at the Laboratory for Synthesis and Analysis of Biomolecules, Institute of Chemistry, University of Brasilia.

The bacterial strains used in this study were Gram-negative species *E. coli* ATCC 25922 and *E. coli* KPC+ HRAN 1812446 (clinical isolate) and Gram-positive species *S. aureus* ATCC 25923 and *S. aureus* MDR LACEN 3730529 (clinical isolate).

### 4.2. Evaluation of the Minimum Inhibitory Concentration (MIC) and Minimum Microbicidal Concentration (MMC) Against Bacteria

MIC susceptibility testing was conducted in accordance with the CLSI protocol M07-A10 [77]; the concentration ranges were 64–0.12 µM for antibiotics, 1024–1 µM for alkaloids, and 128–0.25 µM for the IAPs. Alkaloid samples were diluted in dimethyl sulfoxide (DMSO), and the final solvent concentration did not exceed 2% (*v*/*v*) of the total well volume (100 µL). Antibiotic and peptide solutions in ultrapure water were diluted in Mueller–Hinton (MH) broth. Bacterial suspensions were adjusted to 5 × 10^5^ CFU/mL and incubated in MH broth in flat-bottom 96-well plates, cell culture-treated (Kasvi, Curitiba, Brazil) (100 μL per well) for 24 h at 37 °C. Bacterial growth in the microplates was recorded by visual inspection of turbidity, with the MIC defined as the lowest concentration at which no visible growth was observed.

Growth controls (medium + bacteria, no compound), inhibition controls (medium + bacteria + reference antibiotic), and sterility controls (medium only) were included. All tests were conducted in technical and biological triplicates. To evaluate potential solvent interference, the MIC and MMC assays were performed on all strains using a DMSO concentration gradient ranging from 32% to 0.25% (*v*/*v*) in a total volume of 100 µL per well.

Following MIC determination, MMC was assessed to confirm bacterial eradication by spotting 10 µL of the supernatant from wells with no visible growth onto MH agar plates. Plates were incubated at 37 °C for 24 h. Colony presence or absence was visually recorded to determine bactericidal concentrations. All tests were conducted in technical and biological triplicates.

### 4.3. Fractional Inhibitory Concentration Index Evaluation

Checkerboard microdilution assays were performed to evaluate synergistic and additive interactions between peptides and alkaloids. Serial dilutions were prepared based on individual MICs. Bacterial suspensions (5 × 10^5^ cells/mL) were incubated in MH broth medium at 37 °C for 24 h in 96-well plates (100 µL/well). Alkaloids were tested at concentrations ranging from 1024 µM to 8 µM, while peptides were evaluated within a range of 8 µM to 0.063 µM. The tests were performed in technical and biological triplicate. The Fractional Inhibitory Concentration Index (FICI) was calculated as follows: FICI = (CA/MIC_A) + (CB/MIC_B), where CA and CB are concentrations in combination, and MIC_A and MIC_B are individual MICs [78]. Combinations with synergistic or additive activity were further evaluated by MMC testing [77]. For alkaloids that did not show an MIC in the tested range, a value of 1024 µM was used. Although this condition does not yield a precise FICI value, we can infer that any combination resulting in a 4× lower peptide concentration is likely synergistic. All tests were conducted in technical and biological triplicates.

### 4.4. Biofilm Prevention Assessment

Biofilm inhibition was assessed using the lowest FICI combination values obtained in the checkerboard assays. Bacterial cultures were first grown on solid media for 20 h at 37 °C. From this growth, a liquid inoculum was prepared by transferring several colonies into 5 mL of the appropriate biofilm growth medium in 50 mL Falcon™ tubes (Kasvi, Curitiba, Brazil). The suspensions were incubated at 37 °C with orbital shaking at 100 rpm for approximately 2 h until the optical density (OD) exceeded 0.1. For *S. aureus* (ATCC 25923) and the multidrug-resistant *S. aureus* clinical isolate (LACEN 3730529), biofilm growth was performed in unmodified MH broth. For *E. coli* (ATCC 25922), biofilm formation was conducted in M63 medium supplemented with 1.5% (*v*/*v*) filtered human blood plasma, 0.4% (*w*/*v*) glucose, and 0.2% (*w*/*v*) casamino acids. The test was then carried out in a cell culture-treated (Kasvi) 96-well plate containing the sterility control, the negative control, and the positive control (gentamicin), as well as the synergistic combinations with the lowest FICI. The plate was incubated at 37 °C for 24 h. After incubation, the culture medium was carefully removed, and wells were rinsed twice with 200 µL of sterile saline. Biofilms were fixed by adding 100 µL of 99% (*v*/*v*) ethanol for 15 min, then removing the ethanol and drying at 37 °C for 15 min. Then, 200 µL of a 0.1% (*v*/*v*) crystal violet solution (in distilled water) was added, and the mixture was incubated for 20 min. Wells were rinsed three times with 200 µL of saline, and the bound dye was solubilized with 200 µL of 30% (*v*/*v*) acetic acid. Absorbance was measured at 600 nm using a spectrophotometer Biotek Power Wave HT (Thermo Fisher, Waltham, MA, USA) [79]. Growth controls (medium + bacteria, no compound), inhibition controls (medium + bacteria + reference antibiotic), and sterility controls (medium only) were included as previously described. Assays were performed in technical and biological triplicate.

### 4.5. Evaluation of the Hemolytic Activity Towards Human Red Blood Cells

Peripheral blood (5 mL) was collected from healthy volunteers (Research Ethics Committee approval #52845021.5.0000.0029) in heparinized collection tubes. The samples were centrifuged at 900× *g* for 10 min, and the plasma was removed. The red blood cells (RBCs) were rinsed three times with PBS (1×, pH 7.4) at room temperature, with centrifugation at 900× *g* between rinses. A 1% (*w*/*v*) RBC solution was prepared from the RBC concentrate. In 96-well microplates, cell culture-treated (Kasvi) test molecules were serially diluted in PBS. Positive control wells contained 0.1% (*v*/*v*) Triton X-100, and negative control wells contained RBCs in PBS only. After setup, 1% (*w*/*v*) RBC suspension was added to each well, bringing the final reaction volume to 160 µL. Plates were incubated at 37 °C for 1 h and centrifuged at 900× g for 5 min. Subsequently, 100 µL of the supernatant was transferred to a new 96-well plate, and absorbance was measured at 540 nm using a spectrophotometer [80]. All assays were conducted in technical and biological triplicates, including blank wells containing PBS only. Hemolysis percentage was calculated as follows: Hemolysis (%) = (Abs_sample/Abs_positive control) × 100 [81,82].

### 4.6. Statistical Analysis

GraphPad Prism Software^®^, version 10.2.3 (San Diego, CA, USA, 2022), was used for the statistical analysis. All tests were performed with at least three biological and technical replicates (SD). First, descriptive statistics were analyzed for the sample to obtain the mean and standard deviation. Any outliers were identified and evaluated using the Grubbs method (95% confidence interval) and removed. Next, the Shapiro–Wilk test for normality was performed at the 95% confidence level. ANOVA was used for normally distributed samples, with Dunnett’s post hoc test for multiple comparisons at the 95% confidence level (*p* < 0.05). When non-normality was observed in the treatment, a nonparametric analysis was performed as appropriate (Kruskal–Wallis test, equivalent to ANOVA, with Dunn’s post hoc test for multiple comparisons, 95% confidence, *p* < 0.05). Finally, representative graphs were generated, and the error bars in the figures represent the SD. This analysis was used to evaluate hemolysis and biofilm prevention.

## 5. Conclusions

The present findings indicate that combinations of IAPs and alkaloids can significantly enhance antimicrobial activity against both ATCC and drug-resistant bacterial strains. Among the evaluated combinations, Hs02 with sinomenine showed particularly strong effects, including marked reductions in biofilm formation and decreases in the concentrations required for bacterial inhibition.

Because tomatidine and sinomenine did not reach measurable MIC values within the tested range, the magnitude of the observed synergistic interactions is likely underestimated. Nevertheless, the corresponding FICI values should be interpreted conservatively. In addition, the hemolysis assay indicated low erythrocyte disruption for the Hs02-alkaloid combinations under the conditions tested, suggesting preliminary hemocompatibility.

Overall, these results suggest that peptide–alkaloid combinations represent promising antimicrobial alternatives. However, further studies are still needed to clarify the mechanisms underlying the observed synergy and to assess cytotoxicity, genotoxicity, inflammatory effects, and in vivo performance.

## Figures and Tables

**Table 1:** Minimum inhibitory concentration (MIC) and minimum microbicidal concentration (MMC) of peptides Hs02 and Gr01, alkaloids, and antibiotics evaluated against *Escherichia coli* and *Staphylococcus aureus*.

Compounds		Organisms							
		*S. aureus* ATCC		*S. aureus* MDR		*E. coli* ATCC		*E. coli* KPC+	
		MIC (μM ± SD)	MMC (μM ± SD)	MIC (μM ± SD)	MMC (μM ± SD)	MIC (μM ± SD)	MMC (μM ± SD)	MIC (μM ± SD)	MMC (μM ± SD)
Peptides	Hs02	8 ± 0.0	8 ± 0.0	2 ± 0.0	2 ± 0.0	4 ± 0.0	4 ± 0.0	4 ± 0.0	4 ± 0.0
	Gr01	4 ± 0.0	8 ± 0.0	4 ± 0.0	8 ± 0.0	4 ± 0.0	8 ± 0.0	4 ± 0.0	8 ± 0.0
Alkaloids	Berberine	1024 ± 0.0	-	512 ± 0.0	-	-	-	-	-
	Tomatidine	-	-	-	-	-	-	-	-
	Sinomenine	-	-	-	-	-	-	-	-
Antibiotics	Gentamicin	0.25 ± 0.0	0.5 ± 0.0	1 ± 0.0	1.33 ± 0.5	1 ± 0.0	1.55 ± 0.52	-	-
	Amikacin	0.5 ± 0.0	3.5 ± 1.84	1 ± 0.0	3.5 ± 1.84	1 ± 0.0	1.55 ± 0.52	16 ± 0.0	32 ± 0.0
	Ampicillin	2 ± 0.0	3.75 ± 1.25	-	-	-	-	-	-

- No MIC was established among the tested concentrations. MICs are higher than the highest tested concentration.

**Table 2:** Synergism between alkaloids and Hs02 against ATCC and clinical isolates, calculation of FICI, and the activity equivalent to the FICI value.

**Organisms ATCC**	**Molecules**											
	**Ber + Hs02**		**FICI**		**Tom + Hs02**		**FICI**		**Sin + Hs02**		**FICI**	
	**MIC (μM ± SD)**				**MIC (μM ± SD)**				**MIC (μM ± SD)**			
*S. aureus*	64 ± 0.0	1 ± 0.0	0.18	S	8 ± 0.0	1 ± 0.0	0.13	S	8 ± 0.0	2 ± 0.0	0.25	S
*E. coli*	8 ± 0.0	2 ± 0.0	0.5	S	8 ± 0.0	2 ± 0.0	0.5	S	8 ± 0.0	1 ± 0.0	0.25	S
**Clinical Isolates**	**Molecules**											
	**Ber + Hs02**		**FICI**		**Tom + Hs02**		**FICI**		**Sin + Hs02**		**FICI**	
	**MIC (μM ± SD)**				**MIC (μM ± SD)**				**MIC (μM ± SD)**			
*S. aureus* MDR	512 ± 0.0	2 ± 0.0	1.5	I	256 ± 0.0	2 ± 0.0	1.25	I	8 ± 0.0	2 ± 0.0	0.5	S
*E. coli* KPC+	8 ± 0.0	4 ± 0.0	1	A	8 ± 0.0	2 ± 0.0	0.5	S	8 ± 0.0	2 ± 0.0	0.5	S

S—synergistic; A—additive; I—indifferent; Ber—berberine; Tom—tomatidine; Sin—sinomenine.

**Table 3:** Minimum microbicidal concentration for synergistic and additive concentrations against ATCC and clinical isolates.

**Organisms ATCC**	**Molecules**					
	**Ber Hs02**		**Tom Hs02**		**Sin Hs02**	
	**FICI**	**MMC**	**FICI**	**MMC**	**FICI**	**MMC**
*S. aureus*	0.18	BS	0.13	BC	0.25	BS
*E. coli*	0.5	BC	0.5	BS	0.25	BC
**Clinical Isolates**	**Molecules**					
	**Ber Hs02**		**Tom Hs02**		**Sin Hs02**	
	**FICI**	**MMC**	**FICI**	**MMC**	**FICI**	**MMC**
*S. aureus* MDR	1	BS	0.75	BS	0.5	BC
*E. coli* KPC+	1	BC	0.5	BC	0.5	BC

BC—bactericidal; BS—bacteriostatic; Ber—berberine; Tom—tomatidine; Sin—sinomenine.

## Data Availability

The data presented in this study are available on request from the corresponding author.

## Abbreviations

The following abbreviations are used in this manuscript:

AMR	Antimicrobial Resistance
ANOVA	Analysis of Variance
CFU	Colony Forming Units
CLSI	Clinical and Laboratory Standards Institute
DMSO	Dimethyl Sulfoxide
FICI	Fractional Inhibitory Concentration Index
LogP	Partition Coefficient (octanol/water)
MMC	Minimum Microbicidal Concentration
MIC	Minimum Inhibitory Concentration
OD	Optical Density
RBC	Red Blood Cells
SD	Standard Deviation
SwissADME	Swiss Institute of Bioinformatics ADME prediction platform
TPSA	Topological Polar Surface Area

## Appendix A

**Table A1:** Synergism between alkaloids and GR01 against ATCC and resistant strains, calculation of FICI, and the activity equivalent to the FICI value.

**ATCC Strains**	**Molecules**											
	**Ber + Gr01**		**FICI**		**Tom + Gr01**		**FICI**		**Sin + Gr01**		**FICI**	
	**MIC (μM ± SD)**				**MIC (μM ± SD)**				**MIC (μM ± SD)**			
*S. aureus*	256 ± 0.0	0.03 ± 0.0	0.25	S	8 ± 0.0	1 ± 0.0	0.13	S	8 ± 0.0	2 ± 0.0	0.25	S
*E. coli*	8 ± 0.0	2 ± 0.0	0.5	A	8 ± 0.0	2 ± 0.0	0.5	A	8 ± 0.0	1 ± 0.0	0.25	S
**Clinical Isolates**	**Molecules**											
	**Ber + Gr01**		**FICI**		**Tom + Gr01**		**FICI**		**Sin + Gr01**		**FICI**	
	**MIC (μM ± SD)**				**MIC (μM ± SD)**				**MIC (μM ± SD)**			
*S. aureus* MDR	256 ± 0.0	0.03 ± 0.0	0.25	S	1024 ± 0.0	0.03 ± 0.0	1.01	I	512 ± 0.0	1 ± 0.0	1	A
*E. coli* KPC+	1024 ± 0.0	6 ± 0.0	1.06	I	8 ± 0.0	2 ± 0.0	0.5	A	8 ± 0.0	2 ± 0.0	0.5	A

S—synergistic; A—additive; I—indifferent; Ber—berberine; Tom—tomatidine; Sin—sinomenine.

## Appendix B

**Table A2:** Statistical table with significance data, statistical difference index, and *p*-value for percentage of hemolysis of the isolated IAPs. Dunnett’s post-test with a 95% confidence interval—*p* < 0.05, after performing the ANOVA test. ****—statistical difference when *p* < 0.0001; ns—not significant.

**Hemolysis IAP Hs02**			
**Dunnett’s Test**	**Significant**	**Index**	***p*-Value**
Triton X-100 0.1% vs. 128 µM	No	ns	0.5633
Triton X-100 0.1% vs. 64 µM	No	ns	>0.9999
Triton X-100 0.1% vs. 32 µM	Yes	****	<0.0001
Triton X-100 0.1% vs. 16 µM	Yes	****	<0.0001
Triton X-100 0.1% vs. 8 µM	Yes	****	<0.0001
Triton X-100 0.1% vs. 4 µM	Yes	****	<0.0001
Triton X-100 0.1% vs. 2 µM	Yes	****	<0.0001
Triton X-100 0.1% vs. 1 µM	Yes	****	<0.0001
Triton X-100 0.1% vs. 0.5 µM	Yes	****	<0.0001
Triton X-100 0.1% vs. 0.25 µM	Yes	****	<0.0001
**Hemolysis IAP Gr01**			
**Dunnett’s Test**	**Significant**	**Index**	***p*-Value**
Triton X-100 0.1% vs. 128 µM	No	ns	>0.9999
Triton X-100 0.1% vs. 64 µM	No	ns	0.8967
Triton X-100 0.1% vs. 32 µM	No	ns	0.2356
Triton X-100 0.1% vs. 16 µM	No	ns	0.9745
Triton X-100 0.1% vs. 8 µM	No	ns	0.977
Triton X-100 0.1% vs. 4 µM	Yes	****	<0.0001
Triton X-100 0.1% vs. 2 µM	Yes	****	<0.0001
Triton X-100 0.1% vs. 1 µM	Yes	****	<0.0001
Triton X-100 0.1% vs. 0.5 µM	Yes	****	<0.0001
Triton X-100 0.1% vs. 0.25 µM	Yes	****	<0.0001

**Table A3:** Statistical significance data, statistical difference index, and *p*-value for percentage of hemolysis of isolated alkaloids according to Dunnett’s test with 95% confidence interval—*p* < 0.05, after performing ANOVA test. ****—statistical difference when *p* < 0.0001.

**Hemolysis Berberine**			
**Dunnett’s Test**	**Significant**	**Index**	***p*-Value**
Triton X-100 0.1% vs. 512 µM	Yes	****	<0.0001
Triton X-100 0.1% vs. 256 µM	Yes	****	<0.0001
Triton X-100 0.1% vs. 128 µM	Yes	****	<0.0001
Triton X-100 0.1% vs. 64 µM	Yes	****	<0.0001
Triton X-100 0.1% vs. 32 µM	Yes	****	<0.0001
Triton X-100 0.1% vs. 16 µM	Yes	****	<0.0001
Triton X-100 0.1% vs. 8 µM	Yes	****	<0.0001
**Hemolysis Sinomenine**			
**Dunnett’s Test**	**Significant**	**Index**	***p*-Value**
Triton X-100 0.1% vs. 512 µM	Yes	****	<0.0001
Triton X-100 0.1% vs. 256 µM	Yes	****	<0.0001
Triton X-100 0.1% vs. 128 µM	Yes	****	<0.0001
Triton X-100 0.1% vs. 64 µM	Yes	****	<0.0001
Triton X-100 0.1% vs. 32 µM	Yes	****	<0.0001
Triton X-100 0.1% vs. 16 µM	Yes	****	<0.0001
Triton X-100 0.1% vs. 8 µM	Yes	****	<0.0001
**Hemolysis Tomatidine**			
**Dunnett’s Test**	**Significant**	**Index**	***p*-Value**
Triton X-100 0.1% vs. 512 µM	Yes	****	<0.0001
Triton X-100 0.1% vs. 256 µM	Yes	****	<0.0001
Triton X-100 0.1% vs. 128 µM	Yes	****	<0.0001
Triton X-100 0.1% vs. 64 µM	Yes	****	<0.0001
Triton X-100 0.1% vs. 32 µM	Yes	****	<0.0001
Triton X-100 0.1% vs. 16 µM	Yes	****	<0.0001
Triton X-100 0.1% vs. 8 µM	Yes	****	<0.0001

**Table A4:** Statistical significance data, statistical difference index, and *p*-value for the percentage of hemolysis of the combinations. Dunnett’s post-test with 95% confidence interval—*p* < 0.05, after performing the ANOVA test. ****—statistical difference when *p* < 0.0001.

**Hemolysis Combinations**			
**Dunnett’s Test**	**Significant**	**Index**	***p*-Value**
Triton X-100 0.1% vs. Ber 8 µM + Hs02 4 µM	Yes	****	<0.0001
Triton X-100 0.1% vs. Tom 8 µM +Hs02 2 µM	Yes	****	<0.0001
Triton X-100 0.1% vs. Sin 8 µM +Hs02 2 µM	Yes	****	<0.0001

## Appendix C

**Table A5:** Significance data, statistical difference index, and *p*-value for biofilm formation across Gram + and − (ATCC and clinical isolates). Dunnett’s post-test with 95% confidence interval—*p* < 0.05, after performing the ANOVA test. **—statistical difference when *p* < 0.01; ***—statistical difference when *p* < 0.001; ****—statistical difference when *p* < 0.0001; ns—not significant.

**Biofilm Formation *S. aureus* ATCC 25923**			
**Dunnett’s Test**	**Significant**	**Index**	***p*-Value**
Negative Control vs. Berberine + Hs02	No	ns	0.4154
Negative Control vs. Sinomenine + Hs02	Yes	****	<0.0001
Negative Control vs. Tomatidine + Hs02	No	ns	0.239
**Biofilm Formation *S. aureus* Multi**			
**Dunnett’s Test**	**Significant**	**Index**	***p*-Value**
Negative Control vs. Berberine + Hs02	Yes	**	0.0019
Negative Control vs. Sinomenine + Hs02	Yes	****	<0.0001
Negative Control vs. Tomatidine + Hs02	No	ns	0.1102
**Biofilm Formation *E. coli* ATCC 25922**			
**Dunnett’s Test**	**Significant**	**Index**	***p*-Value**
Negative Control vs. Berberine + Hs02	Yes	***	0.0002
Negative Control vs. Sinomenine + Hs02	Yes	****	<0.0001
Negative Control vs. Tomatidine + Hs02	Yes	**	0.0086
